# Use of Continuous Positive Airway Pressure Ventilation as a Support During Coronary Angioplasty in Patients with Acute Myocardial Infarction: Safety and Feasibility

**DOI:** 10.3390/jcm14165756

**Published:** 2025-08-14

**Authors:** Francesca Giordana, Filippo Angelini, Marisa Gribaudo, Giorgio Baralis, Sebastian Andrea Cinconze, Mauro De Benedetto Fabrizi, Cristina Battaglia, Andrea De Stefanis, Allison Verra, Roberta Rossini

**Affiliations:** 1 Division of Cardiology, Santa Croce and Carle Hospital, 12100 Cuneo, Italy; gribaudo.m@ospedale.cuneo.it (M.G.); baralis.g@ospedale.cuneo.it (G.B.); debenedettofabrizi.m@ospedale.cuneo.it (M.D.B.F.); battaglia.c@ospedale.cuneo.it (C.B.); rossini.r@ospedale.cuneo.it (R.R.); 2 Division of Cardiology, Cardiovascular and Thoracic Department, Città della Salute e della Scienza, 10126 Turin, Italy; filippoangelini90@gmail.com; 3 Divisione di Cardiologia, Ospedale Civile di Vigevano, 27029 Vigevano, Italy; cinconze.s@gmail.com; 4 Division of Cardiology, Department of Medical Sciences, Città della Salute e della Scienza Hospital, University of Turin, 10124 Turin, Italy; andredeste1997@gmail.com (A.D.S.); ally.verra@gmail.com (A.V.)

**Keywords:** non-invasive ventilation, coronary disease, heart failure

## Abstract

**Background/Objectives**: To evaluate the safety and feasibility of continuous positive airway pressure (CPAP) in patients with acute myocardial infarction (AMI) and acute decompensated heart failure (ADHF) during percutaneous coronary intervention (PCI). Non-invasive ventilation (NIV) is an established treatment for ADHF. **Methods**: All consecutive patients admitted to Santa Croce Hospital of Cuneo, receiving CPAP for ADHF in the cath lab during PCI for AMI, were included in a case series. **Results**: Between December 2018 and March 2021, 25 pts were included (median age 78 yrs, 48% female), with 64% of patients presenting with ST-elevation AMI and 17 (69%) in cardiogenic shock. At admission median left ventricular ejection fraction was 35 (20–60)% and eight (32%) patients had severe mitral regurgitation. Median PaO_2_/FiO_2_ was 183 (141–261) mmHg/%, lactate level 2.4 (1.3–3.8) mmol/L, and NTproBNP 7882 (3139–35,000) ng/L. CPAP was positioned and managed by nurses in all cases. Median FiO_2_ was 50 (35–100)% and median positive end-expiratory pressure was 7.5 (5–12) cmH_2_O. CPAP was generally well tolerated in 22 (88%) patients. One patient suffered cardiac arrest that led to CPAP interruption due to resuscitation maneuvers. No patient required orotracheal intubation in the cath lab. The post-procedural PaO_2_/FiO_2_ ratio substantially improved to 230 (175–356) mmHg/% (*p* = 0.007) and lactate decreased to 1.5 (1.0–1) mmol/L (*p* = 0.002). One patient died during hospital stay due to underlying disease, unrelated to the study procedure. **Conclusions**: CPAP during PCI in patients with AMI and ADHF seems feasible, safe, and well tolerated. Larger studies are warranted to confirm these results.

## 1. Introduction

Incidence of acute decompensated heart failure (ADHF) among patients admitted with an acute myocardial infarction (AMI) varies among studies, ranging from 14% to 36% [1]. The FAST-MI registry showed that AMI patients with ADHF, compared with AMI patients without ADHF, have a significantly increased risk of death during index hospitalization (12.2% vs. 1.3%; *p* < 0.0001) and at one year of follow-up (26.6% vs. 5.2%; *p* < 0.0001) [2]. A recent study demonstrated that one out of three patients presenting with a high Killip class died in the first year after index hospitalization, regardless of the type of acute coronary syndrome [3]. Furthermore, the authors observed a proportional correlation between the degree of Killip class (II-III-IV) and adverse prognosis. These data suggest that AMI patients’ prognosis is closely linked to clinical presentation and, in particular, to Killip class. This implies that prompt treatment not only of coronary artery disease but also of ADHF is warranted in each patient. Greater efforts should be focused on improving the use of therapies recommended by the guidelines in a prompt and effective manner on the one hand and on finding new specific and timely treatments in this high-risk population on the other [3]. A Danish registry identifies cardiogenic shock, other than cerebral anoxia post-cardiac arrest, as main causes of death at 30 days [4]. Many strategies have been implemented to prevent cardiogenic shock, based on increasing mechanical circulatory support accessibility (IABP and VA-ECMO) [5]. These devices can be useful in AMI complicated with cardiogenic shock to left ventricular unloading and to maintain an adequate cardiac output, even during hemodynamic study and coronary angioplasty procedures. The main weakness of these devices lies in their cost, which makes them difficult to implement on a large scale [6,7]. Patients with non-ST-elevation myocardial infarction (NSTEMI) tend to develop acute heart failure later than those with ST-elevation myocardial infarction (STEMI), providing a wider time window for the prevention of cardiogenic shock [8]. Current guidelines offer clear recommendations regarding the management of these patients—namely, early hemodynamic assessment and the potential need for mechanical circulatory support; however, they do not specify which type of ventilatory support should be preferred for managing acute respiratory failure, which is frequently associated with these hemodynamic deterioration patterns, especially during hemodynamic procedures [9]. Non-invasive mechanical ventilation has proven to be a useful tool in the setting of acute respiratory failure in ADHF, as it reduces the need for orotracheal intubation (OTI) and decreases the associated risk of infections, mainly ventilator-associated pneumonia [10]. Acute pulmonary edema is the second leading cause of acute respiratory failure after pneumonia [11]. A variable degree of pulmonary edema (interstitial and/or alveolar) is observed in the majority of patients with acute heart failure: nearly 90% of patients report dyspnea, yet fewer than half show abnormalities in arterial blood gas analysis, such as hypoxemia, hypercapnia, acidosis, or a combination of these [12]. Several studies have demonstrated that early application of continuous positive airway pressure (CPAP) in patients with acute cardiogenic pulmonary edema in the pre-hospital setting leads to a more rapid improvement in respiratory failure compared to conventional oxygen therapy (OT), with a tendency to reduce the rate of OTI [13]. Moreover, CPAP is readily accessible, as it does not require specialized training or expensive equipment. There are two main modes of non-invasive ventilation (NIV): CPAP and NIPSV (non-invasive pressure support ventilation with positive end-expiratory pressure); both of them have proven to be effective in the treatment of acute respiratory failure of cardiogenic origin, showing a reduction in mortality compared to oxygen therapy alone, with a statistically significant effect observed for CPAP [14]. Thanks to the possibility of applying positive pressure even in unconscious patients through the use of various interfaces, non-invasive ventilation gains a relevant role in many care settings, ranging from pre-hospital treatment to home care, whereas IOT has remained limited to critical care units or the operating room. In the setting of ADHF due to an AMI, the hemodynamic effects of CPAP, which are the reduction in pre-load and after-load, may help to increase cardiac output [15]. Data on CPAP use in the cath lab in the setting of AMI during percutaneous coronary intervention are lacking. The latest ESC guidelines recommend NIV as a Class IIa, level of evidence B indication in patients with respiratory distress (respiratory rate > 25 breaths/min, SpO_2_ < 90%) [16]. The NICE guidelines recommend NIV in patients with acute cardiogenic pulmonary edema (ACPE) presenting with severe dyspnea and acidosis [17]. Finally, the most recent ERC/ATS guidelines advocate for the use of NIV—both BiLevel and CPAP—in this setting, starting already in the pre-hospital phase [18]. The aim of the present case series is to evaluate safety and feasibility of CPAP ventilation during PCI in patients admitted for AMI and presenting ADHF.

## 2. Materials and Methods

The medical records of patients admitted to the Cardiology Department at Santa Croce e Carle Hospital, Cuneo, Italy, between December 2018 and March 2021 were retrospectively reviewed. Patients were included if they met all of the following criteria: diagnosis of AMI—either STEMI or NSTEMI—at admission; clinical signs and symptoms consistent with ADHF—defined as new or worsening dyspnea associated with pulmonary congestion on chest X-ray, elevated natriuretic peptides, and/or signs of volume overload [19]; evidence of acute respiratory failure based on arterial blood gas analysis; and administration of CPAP ventilation during percutaneous coronary intervention (PCI) as prescribed by the attending physician. Patients were excluded if they received medical therapy without undergoing PCI or if CPAP ventilation was not utilized during the procedure.

Data extraction was performed by two independent cardiologists (FA and AD) using a standardized data collection form. Cases with missing arterial blood gas measurements before or after PCI were excluded from the respective analysis. No imputation was performed.

Demographic characteristics, comorbidities, clinical presentation, laboratory findings, echocardiographic parameters, CPAP settings, relevant procedural features of PCI, length of hospital stay, and arterial blood gas values were collected. Specifically, the first arterial blood gas sample obtained prior to coronary angiography and the first one collected following revascularization were recorded.

Prior to the adoption of CPAP use during PCI, a dedicated training program was conducted for all nurses working in the catheterization laboratory. The nurse training program consisted of a 2 h theoretical session on the use of CPAP, followed by a regular clinical in the ICU, where competency was confirmed via direct observation by expert operators.

The CPAP system utilized was MYO—MODD. MTMYO-01^®^ (MEDIVAL S.R.L., Padua, Italy) with a flow generator capable of delivering pressures from 5 to 15 cm H_2_O. Disposable total face masks (MEIKA group, Mirandola, Italy) were used to minimize air leakage and patient discomfort.

CPAP was typically initiated once the patient’s systemic blood pressure was stabilized. An initial low level of positive end-expiratory pressure (PEEP) was applied to improve patient tolerance of the mask and then titrated according to the judgment of the treating physician [20]. CPAP settings were reassessed at 5 min intervals during PCI. FiO_2_ levels were initially set at 0.5 and adjusted based on pulse oximetry and arterial blood gas measurements to maintain SpO_2_ ≥ 94%.

### 2.1. Study Endpoints

The primary endpoint was the feasibility of CPAP application during PCI, defined as the successful initiation and management of CPAP within the catheterization laboratory during the procedure. Patient tolerance to CPAP therapy was also documented. Secondary endpoints included changes in arterial blood gas parameters, requirement for anesthesiology support, need for OTI, in-hospital mortality, 30-day mortality, and length of stay in the intensive care unit (ICU) and overall hospital stay.

### 2.2. Statistical Analysis

Categorical variables are presented as counts and percentages. Continuous variables are expressed as mean ± standard deviation or as median with interquartile range (IQR), as appropriate. Normality of distribution was assessed using the Shapiro–Wilk test, and homogeneity of variances was evaluated with Levene’s test prior to applying parametric analyses. Comparisons between groups were conducted using Student’s *t*-test for normally distributed variables and the Wilcoxon rank-sum test for non-normally distributed data. For categorical data, the Chi-square test or Fisher’s exact test was used based on expected frequencies. Missing data were handled using listwise deletion, and the proportion of missing values for each variable was reported. Effect sizes were calculated for statistically significant comparisons to provide clinical relevance, and 95% confidence intervals were presented where applicable. Exploratory subgroup analyses were also conducted to assess potential interactions between baseline variables and clinical outcomes, although these were not adjusted for multiple comparisons. A two-sided *p*-value < 0.05 was considered statistically significant. Statistical analyses were performed using SPSS version 26.0 (SPSS Inc., Chicago, IL, USA), and the statistical plan was reviewed a priori by an independent statistician to ensure methodological rigor. The study protocol was approved by the local Ethics Committee. Due to the retrospective nature of this study, the requirement for written informed consent was waived. All data were anonymized prior to analysis, and the study was conducted in accordance with the Declaration of Helsinki. The data supporting the findings of this study are available from the corresponding author upon reasonable request.

## 3. Results

Between December 2018 and March 2021, 25 patients were admitted with a diagnosis of AMI and symptoms of ADHF and respiratory failure and were treated with CPAP during PCI. Baseline characteristics of these patients are reported in Table 1.

The median age was 78 yrs (IQR 70–86) and 12 (48%) were female. Most of them had a history of hypertension (72%). The most common presentation was STEMI (64%); 15 patients were classified as Killip III class while 9 patients were classified as Killip IV class.

Among patients with STEMI, 13 (81%) had anterior infarctions and 3 (19%) had inferior infarctions, 1 of whom presented with right ventricular involvement. The mean peak troponin level was 115,814 ng/L (IQR: 10,816–113,754), suggesting a substantial infarct size across the population. Mean systolic blood pressure at presentation was 130 ± 30 mmHg. Mean ejection fraction at admission was 35 ± 10%, and one out of three patients had moderate to severe mitral regurgitation. According to the Society for Cardiac Angiography and Intervention (SCAI) classification [21], 2 (8%) were classified as grade A, 10 (40%) grade B, 11 (44%) C, and 2 (8%) grade D.

Median PCI duration was 56 min (IQR 40–81), with 54 min (IQR 20–140) for STEMI and 58 min (IQR 20–90) for NSTEMI patients. PCI was performed via the radial approach in 64% of patients. Inotropic and/or vasopressor support was necessary in 13 (52%) cases, with an intra-aortic balloon pump (IABP) support being required in 7 (28%) patients.

CPAP was positioned and managed by nurses in all cases. Median FiO_2_ was 50 (IQR 35–100)%, with a mean PEEP of 7.5 (5–12) cmH_2_O.

CPAP was generally well tolerated in 22 (88%) patients. CPAP was associated with a significantly higher oxygen concentration (59 vs. 114 mmHg, *p* < 0.001) and pO_2_/FiO_2_ (183 vs. 230, *p*-value 0.007) with significant lactate clearance (2.4 to 1.5 mmol/L, *p*-value 0.002) (Table 2).

One patient with an acute bi-ventricular disfunction, due to the involvement of the right ventricle in an inferior STEMI, well tolerated a low end-expiratory positive pressure (up to 7.5 mmHg) as well, with prompt and significant lactate level clearance (from 4.9 to 1.6 mmol/L).

In a small proportion of cases (16%), anesthesiologist intervention was required (Table 3).

There were no cases of OTI during PCI, but in a single case OTI was deemed necessary during the index hospitalization, due to an acquired pneumonia. Median ICU hospitalization was 7 days (IQR 5–10), while total hospital stay was 10 days [7,8,9,10,11,12,13,14]. One patient died during hospitalization due to underlying disease, unrelated to the study procedure, and no further deaths occurred at a 30-day follow-up (Table 4).

## 4. Discussion

In this retrospective case series, we report the feasibility of non-invasive ventilation with CPAP in the cath lab in AMI patients undergoing PCI presenting with signs and symptoms of ADHF. A significant portion (69%) of patients presented with cardiogenic shock. All patients received optimal medical therapy and underwent an invasive strategy according to international guidelines [9,19,21].

Although CPAP has been approved for the treatment of ADHF—even when progressed to cardiogenic shock [22]—in intensive, sub-intensive, and emergency settings, data regarding its use specifically in the cath lab remains limited. Likewise, evidence on its safety in AMI patients with ADHF is scarce.

Our findings show that non-invasive ventilatory support with CPAP during PCI in patients with AMI and respiratory failure is feasible, with a high success rate in placement and maintenance and no need for anesthesiologic support. The majority of patients (88%) tolerated the mask well, and no OTI was required, compared to a 4% intubation rate reported by a recent meta-analysis on CPAP in emergency settings [23].

The recent European heart failure guidelines [19] emphasize early initiation of non-invasive ventilation in patients with respiratory distress to improve gas exchange and reduce both the need for OTI [16,24] and the incidence of ventilator-associated pneumonia [22].

CPAP was generally well tolerated without causing hemodynamic compromise. Most patients had advanced Killip class and moderate left ventricular ejection fraction reduction; half required inotropes and one-third required IABP. A recent analysis on AMI patients with cardiogenic shock using the SCAI classification [21] showed a survival rate of 76% in shock stages C and D, 58% in stage E, and below 20% for patients classified as stage E after 24 h [25].

The potential benefits of CPAP in AMI with ADHF were first suggested in 1985 [26]. CPAP enhances tissue oxygenation, reduces interstitial edema, improves pulmonary ventilation, and decreases left ventricular transmural pressure and oxygen consumption—key mechanisms supporting myocardial recovery. These effects may reduce the need for diuretics and limit kidney damage from hypoperfusion or contrast-induced nephropathy [27].

However, positive pressure ventilation must be used cautiously in conditions like acute pulmonary hypertension, right ventricular failure, or tamponade, as it may impair right ventricular function [28]. To minimize hemodynamic risks, CPAP was initiated after pressure stabilization with a low initial PEEP. Even a patient with biventricular dysfunction tolerated low PEEP well, showing significant lactate clearance.

Among the 22 patients who tolerated CPAP during PCI, arterial blood gases showed an improved PaO_2_ and P/F ratio, contributing to lactate reduction, as shown in prior studies [29]. Wang et al. demonstrated that CPAP not only improved oxygenation in hypoxic AMI patients but also enhanced hemorheological properties by increasing erythrocyte deformability and reducing aggregation—factors that lower blood viscosity and improve hemodynamics [30].

- Importantly, CPAP did not prolong PCI duration; on the contrary, it may have facilitated procedures by improving respiratory function and patients’ tolerance of the supine position.
- Recent advances in pre-hospital care have emphasized the early initiation of NIV, especially in cases of acute cardiogenic pulmonary edema, to avoid the progression of respiratory failure before hospital admission [31]. Several emergency medical systems now incorporate CPAP devices in ambulances, allowing stabilization of patients en route to definitive care facilities [23].
- This proactive approach aligns with growing evidence from randomized controlled trials suggesting that early NIV application in acute heart failure syndromes significantly reduces the incidence of orotracheal intubation and associated complications, such as ventilator-associated pneumonia and sedation-related delirium.
- Furthermore, data from multicenter registries have indicated that the adoption of non-invasive ventilatory strategies is associated with a reduction in in-hospital mortality and ICU length of stay, reinforcing the notion that early respiratory support should be integral to the acute coronary syndrome care pathway [14].
- In our study, the favorable safety profile of CPAP may also be attributed to careful patient selection and stepwise titration protocols that minimized hemodynamic instability. Protocol-driven application of CPAP, supported by nursing staff trained specifically in cath lab respiratory management, likely contributed to the high tolerance observed.
- The involvement of trained nurses in the management of CPAP during PCI reflects a growing trend toward task-sharing in cardiovascular interventions, where multidisciplinary collaboration has been shown to improve patient safety and procedural efficiency.
- Several studies have advocated for structured training programs for nursing and allied healthcare personnel involved in acute cardiac care, particularly in non-invasive respiratory support, highlighting improvements in care quality and reductions in intervention-related complications [32].
- Moreover, novel CPAP interface technologies, such as helmet CPAP, have emerged as potentially superior alternatives to traditional face masks, offering enhanced comfort, better sealing, and reduced aerosol dispersion—important considerations in contemporary clinical environments affected by infectious respiratory diseases [33].
- As patient-centered care becomes increasingly emphasized, attention to tolerability and device ergonomics may play a role in improving adherence and outcomes in both emergency and prolonged applications of non-invasive ventilation.
- It is also noteworthy that our findings support the feasibility of CPAP without anesthesiologic supervision during PCI—a relevant aspect in resource-limited or high-volume settings where immediate access to anesthesiology services may not be guaranteed.
- This independence further underscores the role of cardiologists and interventional teams in managing acute respiratory compromise with non-invasive methods, facilitating uninterrupted procedural workflows.
- However, future studies are needed to better define patient subgroups who might benefit most from this strategy. Stratification by biomarkers, echocardiographic profiles, or shock severity scores could help tailor respiratory support and maximize therapeutic benefit.
- The integration of CPAP into cath lab protocols should be studied not only in terms of feasibility and safety, but also for its potential impact on longer-term outcomes, including 30-day mortality, readmission rates, and functional recovery.

## 5. Limitations

The main limitations of the present case series are the limited number of patients and the retrospective and monocentric nature of this study. The use of CPAP was left at the discretion of the treating physician, potentially introducing a selection bias. Moreover, the first available arterial blood gas analysis was sometimes collected after the initiation of oxygen support in the emergency department, thus potentially leading to an underestimation of respiratory compromise at presentation. In addition, the small sample size did not allow multivariate analyses of the data, and the absence of a control group limits the strength of causal inference.

Another notable limitation is the lack of standardized timing for CPAP initiation, which introduces heterogeneity in treatment exposure. In some cases, CPAP may have been applied early in the emergency department, while in others, it was initiated only upon arrival in the cath lab. This variability may have influenced outcomes and should be addressed in future studies through protocol-driven strategies. Furthermore, the absence of comparator groups receiving standard oxygen therapy alone prevents a direct evaluation of the incremental benefit of CPAP in this setting.

Additionally, echocardiographic data or hemodynamic parameters such as pulmonary capillary wedge pressure or cardiac output were not systematically collected. These measurements would have allowed for a more accurate assessment of heart failure severity and a clearer understanding of CPAP’s physiological effects. The retrospective data collection also precluded follow-up analyses beyond hospital discharge, such as 30-day mortality or readmission rates, which are crucial to evaluate long-term outcomes.

Patient-centered aspects were also not assessed, including CPAP tolerance, comfort, and interface-related complications such as claustrophobia or skin breakdown. These are particularly relevant in the cath lab setting, where procedural anxiety and limited mobility may compromise interface adherence. As the use of CPAP expands into procedural environments, these qualitative parameters should be considered in future research.

## 6. Conclusions

Non-invasive mechanical ventilation with CPAP during PCI, in the setting of AMI complicated by ADHF, seems feasible and safe, being well tolerated during the entire procedure. It can be easily managed by nursing staff without the need for anesthesiologic support in the vast majority of patients. The use of CPAP is associated with a significantly higher PaO_2_ and pO_2_/FiO_2_ with significant lactate clearance.

Cost-effectiveness analyses are warranted to evaluate whether the use of CPAP in this setting leads to reductions in ICU utilization, hospital length of stay, or downstream healthcare resource consumption [34].

Additionally, collaborative registries and multicenter prospective trials are essential to validate these findings and establish standardized guidelines for the use of CPAP during coronary revascularization in the setting of acute heart failure.

## 7. Future Perspectives

Emerging advances in catheter laboratory technology may allow the integration of continuous respiratory monitoring (e.g., respiratory rate, tidal volume, and dynamic compliance) directly with procedural data, enabling real-time adjustments of CPAP settings based on physiologic changes during PCI. Such closed-loop systems could improve safety and efficacy, especially in high-risk patients with dynamic hemodynamic instability.

Furthermore, the deployment of artificial intelligence (AI) algorithms holds promising potential to enhance the application of CPAP in this context. Machine learning models could be trained on multimodal peri-procedural data (e.g., intraprocedural hemodynamics, oxygenation trends, and ventilation parameters) to predict who might benefit most from CPAP, identify early signs of respiratory compromise, and suggest optimal CPAP titration strategies. AI systems working in parallel with the cath lab team could provide decision support, alerting operators to subtle patterns indicative of impending decompensation [35].

Recent research has explored AI in respiratory care, including mechanical ventilation, showing that hybrid ventilator modes driven by intelligent algorithms can reduce the duration of mechanical support and ventilator-associated complications. Translating similar AI-driven decision support to non-invasive ventilation during PCI—even with standard CPAP—could yield comparable benefits [36].

Moreover, future implementations could include wearable or sensor-integrated interfaces (e.g., smart masks) capable of feeding continuous respiratory and cardiovascular data to predictive models, enabling early detection of mask intolerance, interface leaks, or patient distress—potentially reducing complication rates and improving procedural flow.

Finally, multicenter prospective trials incorporating AI-supported CPAP deployment protocols could assess not only safety and procedural outcomes but also longer-term endpoints such as functional recovery, readmission rates, and cost-effectiveness. The convergence of interventional cardiology, respiratory monitoring, and AI-based analytics may thus redefine acute care workflows in AMI complicated by acute decompensated heart failure.

## Figures and Tables

**Table 1:** Baseline characteristics of the study population.

History	N = 25
Age [years, IQR]	78 (70–86)
Female sex (%)	12 (48)
BMI	25 ± 4
Hypertension (%)	18 (72)
Diabetes (%)	7 (28)
Dyslipidemia (%)	13 (52)
COPD (%)	2 (8)
Smoke (%)	8 (32)
History of CAD (%)	5 (20)
**Presentation**	
STEMI (%)	16 (64)
**-anterior**	13 (81)
**-inferior**	3 (19)
NSTEMI (%)	9 (36)
Killip class (%)	
II	1 (4)
III	15 (60)
IV	9 (36)
**Troponin peak [ng/L]**	115,814 (10,816–113,754)
Ejection fraction (%)	35 ± 10
Moderate–severe MR (%)	8/24 (32)
Systolic blood pressure [mmHg]	130 ± 30
Diastolic blood pressure [mmHg]	80 ± 18
Heart rate [bpm]	100 ± 20
Hemoglobin [mg/dL]	14.6 ± 2.7
eGFR [mL/min]	42 (35–71)
NT-proBNP [pg/mL, IQR]	9454 (4588–27,036)
SCAI classification (%)	
SCAI A	2/25 (8)
SCAI B	10/25 (40)
SCAI C	11/25 (44)
SCAI D	2/25 (8)

Abbreviations: BMI: body mass index; CAD: coronary artery disease; COPD: chronic obstructive pulmonary disease, eGFR: estimated glomerular filtration rate; MR: mitral regurgitation; (N)STEMI: (non)ST-elevation myocardial infarction; SCAI: Society for Cardiac Angiography and Interventions.

**Table 2:** Effects of continuous positive airway pressure on arterial blood gas and on blood pressure.

	Baseline	After PCI	*p*-Value
pH (SD)	7.35 ± 0.12	7.37 ± 0.08	0.095
pO_2_ [mmHg, IQR]	59 (50–75)	114 (90–172)	**<0.001**
pCO_2_ [mmHg, IQR]	34 (28–47)	40 (33–47)	0.334
pO_2_/FiO_2_ (IQR)	183 (141–261)	230 (175–356)	**0.007**
HCO_3_^−^ [mmol/L, IQR]	21 (17–24)	23 (20–25)	0.334
Lactate [mmol/L, IQR]	2.4 (1.3–3.8)	1.5 (1.0–1)	**0.002**
Systolic blood pressure [mmHg, SD]	124 ± 28	133 ± 24	0.107
Diastolic blood pressure [mmHg, SD]	77 ± 18	71 ± 15	0.530

**Table 3:** Continuous positive airway pressure and procedural characteristics in the cath lab.

Procedure	N = 25
Duration [min, IQR]	56 (40–81)
Radial access (%)	16 (64)
IABP (%)	7 (28)
CPAP	
FiO_2_ [%, SD]	53 ± 13
PEEP [mmHg, SD]	7.5 ± 2.0
Need for inotropes (%)	13 (52)
Need for vasodilators (%)	11 (44)
Anesthesiologist intervention (%)	4 (16)

Abbreviations: CPAP: continuous positive airway pressure, IABP: intra-aortic balloon pump, PEEP: positive end-expiratory pressure.

**Table 4:** Feasibility and safety outcomes of the study.

Outcomes	N = 25
Compliance to CPAP (%)	22 (88)
Nurse management (%)	25 (100)
OTI during procedure (%)	0 (0)
OTI during hospitalization (%)	1 (4)
Death in-hospital (%)	1 (4)
Death at 30 days (%)	1 (4)
Acute kidney failure (%)	8 (32)
ICU hospitalization length [days, IQR]	7 (5–10)
Total hospitalization [days, IQR]	10 (7–14)

Abbreviations: CPAP: continuous positive airway pressure, ICU: intensive care unit, OTI: orotracheal intubation.

## Data Availability

All data were anonymized prior to analysis, and the study was conducted in accordance with the Declaration of Helsinki. The data supporting the findings of this study are available from the corresponding author upon reasonable request.
